# Mr. Taylor, on Cold Water in Gout

**Published:** 1804-04-01

**Authors:** Thomas Taylor

**Affiliations:** Surgeon. Harleston, Norfolk


### *To the Editors of the Medical and Physical Journal.*

Gentlemen,

THE subject of Gout having lately been brought forward in the Medical and Physical Journal, in consequence of the treatment proposed for its cure by Dr. Kinglake, I shall make no apology for troubling you with the following observations. It is not my intention to enter into the controversy that has taken place between Dr. Kinglake and a Constant Reader, but merely to state facts as they have occurred to my knowledge. The application of cold water, with a view of reducing morbid excess of temperature, does not appear to be a new remedy, but on the contrary; and, like many other remedies for disease, has been laid aside, and after a time resumed.

I have always been in the habit, since I began medical practice, of using cold water as a topical application in local inflammation of the joints; but since Dr. Kinglake's communications, I have employed it in every case of acute rheumatism, as well as gout, which has fallen under my care, and am happy to say with uniform success, in cutting short the disease. Many instances could be produced by me to prove, that the application of cold water is not only only a perfectly safe remedy in gout and rheumatism, but a highly beneficial one. The following is a case of acute rheumatism, in which it was used with decided advantage.

J. Stockdale, aged twenty-three years, was attacked in January last with severe febrile symptoms, pain and swelling in the ancles, knees, and wrists; the pain was very violent, particularly at night, and when in bed. He had been ill a week when I saw him, and had been attacked on the day previous to my visit with a violent fixed pain under the left breast, extending to the clavicle, attended with cough and difficulty in breathing; he expectorated a considerable quantity of bloody mucus. He was ordered to take ten drops of the tinctura digitalis every four hours, in a glass of cold water, and fifteen grains of pulv. ipecac. comp. at night at bed-time; the joints were directed to be kept constantly wet with cloths, dipped in a lotion composed of water, coloured with tinct. lavendulæ composita. On visiting him the next day, I found he had rested better, was freer from pain and the swelling somewhat diminished, but, as his breathing was still impeded and cough troublesome, it was deemed proper to apply a blister to the side. The tinct. digitalis, and pulv. ipecac. comp. were continued as before, and the red lotion applied to the tumefied parts, which he was eagerly desirous of having renewed every few minutes, as it made him so comfortable and easy. He went on mending rapidly, and in the course of seven or eight days from my first seeing him, the swelling and pain had subsided, his fever and cough had left him, and he had not a symptom of disease but general debility, for which he took half a drachm of bark three times a day. It is to be remarked that this patient had been seized with this disease about six weeks before, in a slight degree, from which he soon recovered, but having imprudently exposed himself to wet, brought on this second attack.

From a variety of cases, in which cold water has by my direction been applied to arthritic inflammation, I shall deem it necessary to state only one, as they would all tend to establish the same point, viz. the efficacy and propriety of the remedy. Mrs. B. between fifty and sixty years of age, has been subject to the attack of gout, in one or both feet, for several years. She has, usually, had two or three fits in the year, and those generally tedious and very painful ones. In last December she was attacked with inflammation and swelling in both feet; the pain was very severe. I saw her the day after the coming on of the fit, and proposed the application of the red lotion; but, as

Mrs.

Mrs. B. had always been in the habit of applying warmth, by flannel, &c. she for some time objected to the application of cold, and it was no easy matter for me to overcome her prejudices; however, she consented, and cloths well wetted were wrapped round the feet and kept constantly upon them; the almost immediate effects of which were, an abatement of the pain, and by continuing the application she was in three days completely cured of the complaint, a little stiffness and debility of the foot only remaining for a few days more. The patient was, as may be readily conceived, highly pleased with the remedy, and not a little surprized at its beneficial influence in so short a period.

